# Engineering a novel light-chain single-domain antibody to enable IgG-format bispecific antibody design

**DOI:** 10.1093/abt/tbaf020

**Published:** 2025-10-17

**Authors:** Mingkai Wang, Qingyuan Xu, Yu Kong, Yuxuan Zhong, Feng Yin, Litong Liu, Zhenlin Yang, Tianlei Ying, Yanling Wu

**Affiliations:** Key Laboratory of Medical Molecular Virology (MOE/NHC/CAMS) and Shanghai Institute of Infectious Disease and Biosecurity, School of Basic Medical Sciences, Department of Pulmonary and Critical Care Medicine, Department of Liver Surgery and Transplantation, Zhongshan Hospital, Fudan University, Shanghai 200032, China; Key Laboratory of Medical Molecular Virology (MOE/NHC/CAMS) and Shanghai Institute of Infectious Disease and Biosecurity, School of Basic Medical Sciences, Department of Pulmonary and Critical Care Medicine, Department of Liver Surgery and Transplantation, Zhongshan Hospital, Fudan University, Shanghai 200032, China; Shanghai Key Laboratory of Lung Inflammation and Injury, Department of Pulmonary Medicine, Zhongshan Hospital, Fudan University, Shanghai 200032, China; Key Laboratory of Medical Molecular Virology (MOE/NHC/CAMS) and Shanghai Institute of Infectious Disease and Biosecurity, School of Basic Medical Sciences, Department of Pulmonary and Critical Care Medicine, Department of Liver Surgery and Transplantation, Zhongshan Hospital, Fudan University, Shanghai 200032, China; Shanghai Engineering Research Center for Synthetic Immunology, Shanghai 200032, China; Key Laboratory of Medical Molecular Virology (MOE/NHC/CAMS) and Shanghai Institute of Infectious Disease and Biosecurity, School of Basic Medical Sciences, Department of Pulmonary and Critical Care Medicine, Department of Liver Surgery and Transplantation, Zhongshan Hospital, Fudan University, Shanghai 200032, China; Key Laboratory of Medical Molecular Virology (MOE/NHC/CAMS) and Shanghai Institute of Infectious Disease and Biosecurity, School of Basic Medical Sciences, Department of Pulmonary and Critical Care Medicine, Department of Liver Surgery and Transplantation, Zhongshan Hospital, Fudan University, Shanghai 200032, China; Shanghai Key Laboratory of Lung Inflammation and Injury, Department of Pulmonary Medicine, Zhongshan Hospital, Fudan University, Shanghai 200032, China; Key Laboratory of Medical Molecular Virology (MOE/NHC/CAMS) and Shanghai Institute of Infectious Disease and Biosecurity, School of Basic Medical Sciences, Department of Pulmonary and Critical Care Medicine, Department of Liver Surgery and Transplantation, Zhongshan Hospital, Fudan University, Shanghai 200032, China; Shanghai Key Laboratory of Lung Inflammation and Injury, Department of Pulmonary Medicine, Zhongshan Hospital, Fudan University, Shanghai 200032, China; Key Laboratory of Medical Molecular Virology (MOE/NHC/CAMS) and Shanghai Institute of Infectious Disease and Biosecurity, School of Basic Medical Sciences, Department of Pulmonary and Critical Care Medicine, Department of Liver Surgery and Transplantation, Zhongshan Hospital, Fudan University, Shanghai 200032, China; Shanghai Engineering Research Center for Synthetic Immunology, Shanghai 200032, China; Key Laboratory of Medical Molecular Virology (MOE/NHC/CAMS) and Shanghai Institute of Infectious Disease and Biosecurity, School of Basic Medical Sciences, Department of Pulmonary and Critical Care Medicine, Department of Liver Surgery and Transplantation, Zhongshan Hospital, Fudan University, Shanghai 200032, China; Shanghai Engineering Research Center for Synthetic Immunology, Shanghai 200032, China

**Keywords:** bispecific antibody, human light chain variable domain, light-chain single-domain antibody, antibody engineering

## Abstract

**Background:**

As one of the most promising classes of next-generation antibody therapeutics, bispecific antibodies (bsAbs) have gained increasing attention owing to their unique dual-targeting mechanisms. However, current bsAb formats often face challenges such as low expression levels, poor homogeneity, and unstable therapeutic efficacy due to their complex structures. Therefore, it is urgent to overcome the current technical limitations and develop novel formats of bsAbs with more stable structures and improved expression efficiency.

**Methods:**

Through rational design and phage display-based screening, we engineered a novel light-chain single-domain antibody (VHHL). Using modular assembly and replacement strategies, the VHHL was reconstituted into conventional immunoglobulin G (IgG)s and the resulting bsAbs were comprehensively characterized by size-exclusion high-performance liquid chromatography, biolayer interferometry binding assay, enzyme-linked immunosorbent assay, and flow cytometry.

**Results:**

A light chain engineering strategy combining complementarity-determining region 3 (CDR3)-grafting with site-directed mutagenesis of CDR1/CDR2 was developed to generate VHHLs. Through phage screening, two mouse CD16-specific VHHL candidates with favorable binding affinities and biophysical properties were identified, and one of which was structurally resolved via X-ray crystallography (3.05 Å resolution). When incorporated into full-length IgGs, the resulting bsAbs retained high structural similarity to natural monoclonal antibodies and maintained dual antigen-binding capabilities through their respective light and heavy chains.

**Conclusions:**

Consequently, this study presents a novel IgG-format bsAb platform enabled by the integration of a rationally designed antigen-binding VHHL, providing a streamlined and versatile strategy for the development of multifunctional antibodies.

**Statement of Significance**

We developed a novel class of light-chain single-domain antibody (VHHL). By incorporating VHHL into full-length IgG, the resulting bispecific antibody (bsAb) retained the structural integrity of native IgG while preserving the independent antigen-binding activities of both the heavy and light chains, offering a simplified and flexible approach for bsAb generation.

## Introduction

Antibody therapeutics, as a cornerstone in the biopharmaceutical field, continue to lead the global biopharmaceutical market [1]. In recent years, with the continuous advancement of antibody engineering, various novel antibody therapeutics have emerged and rapidly gained a foothold in the biopharmaceutical market. These next-generation antibody drugs are characterized by higher efficiency, improved specificity, and enhanced safety [2]. Among them, bispecific antibody (bsAb) drugs have become one of the fastest-growing and most widely used categories due to their unique dual-target mechanism. Since the approval of the world’s first bsAb drug, catumaxomab, in 2009, more than a dozen bsAbs have launched to the market and exhibited superior therapeutic potential in tumors, genetic disorders, and autoimmune diseases [3–5]. However, current bsAb formats often face challenges such as low expression levels, poor homogeneity, and unstable therapeutic efficacy due to their complex structures. Therefore, it is urgent to overcome the current technical limitations and develop novel formats of bsAbs with more stable structures and improved expression efficiency, thereby opening up new paradigms and providing solutions for multi-target synergistic therapies.

The antigen-binding specificity of traditional monoclonal antibody (mAb) is endowed by the antigen-binding region composed of the heavy chain variable region (VH) and the light chain variable region (VL), which are structurally interdependent. When either variable region is expressed individually, it typically exhibits poor structural stability and a strong aggregation tendency. In previous studies, our group innovatively developed a modular recombination strategy that divides the human VH domains into framework elements and diversity elements [6]. By systematically screening and reconstructing frameworks from various heavy chain subfamilies, we identified an optimized scaffold with superior stability and solubility. Grafting B cell-derived diversity elements onto this scaffold led to the generation of a novel antibody format, termed the fully human single-domain antibody (UdAb). By screening a large UdAb phage display library, we obtained a series of candidate molecules with high affinities and excellent biophysical properties toward different antigens [7–10]. Based on the above foundation, we further hypothesize that the human VL domain can also be engineered into a functional, antigen-binding single-domain format. Notably, several studies have suggested that human VL generally exhibit reduced aggregation compared to VH, potentially making them more suitable for therapeutic use [11, 12]. Furthermore, reconstituting such single-domain VL within a conventional IgG framework, while preserving their independent antigen-binding function in combination with a heavy chain targeting a different epitope, provides a novel strategy for the generation of bsAbs with native IgG architectures. However, currently identified antigen-binding VL fragments are largely limited by low binding affinity, typically in the range of several hundred nanomolar to micromolar levels, which poses significant challenges for subsequent functional validation and therapeutic development [11, 13, 14]. Additionally, these VL molecules have been independently identified using diverse human light chain subfamilies as scaffolds, resulting in substantial heterogeneity in stability, solubility, and other physicochemical properties [12, 15–17]. Therefore, a systematic approach to develop a well-defined and stable VL scaffold with robust antigen-binding potential could offer a novel and generalizable platform for the construction of IgG-format bsAbs, providing new opportunities for multi-target therapies with improved developability.

In this study, through rational design and phage display-based screening, we generated a novel class of recombinant light-chain single-domain antibodies named VHHLs and identified two promising candidates, L6 and L15, which demonstrated specific binding to mCD16 and favorable biophysical properties. To investigate the structural basis of their stability, we determined the crystal structure of L6 using X-ray crystallography. Furthermore, guided by modular design principles, we reassembled the VHHL into full-length IgG. This novel type of reconstituted antibody retained the structural integrity of native IgG while preserving the independent antigen-binding activities of both the heavy and light chains, thus offering a simplified and flexible approach for bsAb generation.

## Materials and methods

### Cell lines

The Expi293 cell line used for the novel IgG-format bsAb expression was purchased from ThermoFisher (Waltham, MA, USA). The human ovarian adenocarcinoma cell line SKOV-3 was purchased from the Cell Bank of the Chinese Academy of Sciences (Shanghai, China). Both cell lines were cultured in standard cell culture media as indicated by the provider at 37°C in a humidified incubator with 5% CO_2_. All cell lines were regularly tested for mycoplasma contamination and authenticated using short tandem repeat profiling to ensure experimental validity.

### Alpaca immunization and VHH amplification

The adult alpaca used for mCD16 immunization was obtained from Nami Biotechnology (Shanxi, China). For each immunization, 400 μg of mCD16 antigen was emulsified with Freund’s adjuvant at a 1:1 ratio and administered via multiple subcutaneous and intradermal injections. Immunizations were performed every 14 days for a total of four injections. After each immunization, 5 ml of blood was collected, and the serum was separated and stored at −80 C. 7 days after the final immunization, 50 ml of blood was collected, and the peripheral blood mononuclear cells (PBMCs) were isolated according to the manufacturer’s instructions. The total RNA was extracted from PBMCs using the RNeasy Mini Kit (QIAGEN, Valencia, CA, USA), and cDNA was synthesized by reverse transcription polymerase chain reaction (RT-PCR) using the FastKing RT Kit (TIANGEN, Beijing, China). For VHH amplification, a two-step PCR approach was conducted. First, the VH + CH2 fragment was amplified in a 50 μl reaction containing Specific Taq Master Mix (Novoprotein, Suzhou, China), 500 pM of each primer, and 5 μg of cDNA for 26 cycles (30 s at 95°C, 1 min at 52°C, and 50 s at 72°C). Next, the VHH fragments were amplified by using Specific Taq Master Mix, 100 pM of each forward primer (F1-F5), 250 pM of each reverse primer (R1-R2), and 0.1 μg of VH + CH2 for 25 cycles (30 s at 95°C, 30 s at 68°C, and 45 s at 72°C) in a 50 μl reaction system. All primers used for VHH amplification are listed in Supplementary Table S1.

### Stepwise assembly of the VHHL fragment

Three complementarity-determining regions (CDRs) of the VHH and framework regions (FRs) of the immunoglobulin kappa variable 3-20 (IGKV3-20) framework were amplified using primers from Supplementary Table S2. PCR was performed in a 50 μl reaction containing Specific Taq Master Mix, 500 pM concentration of forward and reverse primers, 0.1 μg of templates for 10 cycles at first (30 s at 94°C, 30 s at 52°C, and 30 s at 72°C), and followed by another 20 cycles (15 s at 94°C, 30 s at 52°C, and 30 s at 72°C). Diversity elements (CDRs) and framework elements (FRs) were assembled stepwise using overlap extension PCR. In the first step, FR1 and CDR1 were joined to generate the FR1-CDR1 fragment (Final-11), while FR3 and CDR3 were fused to obtain the FR3-CDR3-FR4 fragment (Final-33). In the second step, CDR2 was combined with the Final-33 fragment to produce the FR2-CDR2-FR3-CDR3-FR4 (Final-23). The Final-11 fragment was further assembled with the Final-23 to produce the VHHL fragment. Except for the final assembly of VHHL, all other amplification steps were performed using a procedure similar to that described previously. The VHHL amplification was carried out using a two-step PCR protocol. The initial amplification was performed in a 90 μl reaction volume containing Specific Taq Master Mix and 0.1 μg each of Final-11 and Final-23, without the addition of primers, for eight cycles (30 s at 94 °C, 30 s at 50 °C, and 1 min at 72 °C). Subsequently, 5 μl of each primer (10 μM) was added, and the amplification was continued for an additional 25 cycles (1 min at 94°C, 1 min at 50°C, and 1 min at 72°C). All primers used in the assembly process are listed in Supplementary Table S3.

### Expression and purification of VHHL

The VHHL fragments were subcloned into the pComb3x vector with N-terminal OmpA signal peptide (MKKTAIAIAVALAGFATVAQA) and C-terminal 6-His tag and Flag tag. The expression of different VHHLs was performed in *Escherichia coli* HB2151 bacterial culture at 30°C for 16 h, accompanied by 1 mM isopropyl β-D-1-thiogalactopyranoside. The cells were harvested and lysed with polymyxin B at 30°C for 0.5 h, followed by centrifugation at 8000 rpm for 10 min. The supernatant was collected, filtered through 0.8 μm polyethersulphone membranes using sterile syringes, and purified by nickel–nitrilotriacetic acid (Ni-NTA) (Smart Lifesciences, Changzhou, China) following the manufacturer’s instructions. Briefly, the filtered supernatant was loaded over the column with Ni-NTA. The resin was washed with washing buffer (10 mM Na_2_HPO_4_, 10 mM NaH_2_PO_4_ [pH 7.4], 500 mM NaCl, and 20 mM imidazole), and proteins were eluted in elution buffer (10 mM Na_2_HPO_4_, 10 mM NaH_2_PO_4_ [pH 7.4], 500 mM NaCl, and 250 mM imidazole). The collected pure fractions were immediately buffer-exchanged into PBS and concentrated with Amicon ultra-centrifugal concentrators (Millipore, Billerica, MA, USA). Protein purity was evaluated by sodium dodecyl sulfate polyacrylamide gel electrophoresis (SDS-PAGE), and the protein concentration was determined using the NanoDrop 2000 spectrophotometer (ThermoFisher). To determine whether the mCD16-specific VHHL retains protein L binding capacity, purification was performed using Capto L resin (GE HealthCare, Marlborough, MA, USA). The purification procedure was similar to that described previously. Firstly, the filtered supernatant was loaded over the column with Capto L. After washing with PBS to remove non-specific proteins, the target proteins were eluted with 0.1 M glycine (pH 3.0) and immediately neutralized with one-tenth volume of 1 M Tris-HCl (pH 9.0). The eluates were concentrated by ultrafiltration and analyzed by SDS-PAGE to assess protein purity.

### Heavy chain CDR amino acid composition analysis

Degenerate primers for CDR1 and CDR2 mutagenesis were designed based on the amino acid frequency distribution of heavy chain CDRs obtained from the AbYsis database [18]. Given the limited number of alpaca antibody entries available and the high similarity between alpaca (*Vicugna pacos*) and human (*Homo sapiens*) CDR composition, frequency data from both alpaca and human antibodies were combined for analysis. For each position, the final amino acid frequency was calculated as the average of the corresponding frequencies in alpaca and human (considering only the 20 types of standard amino acids).

### VHHL library construction

For the construction of the VHHL phage display library against mCD16, the CDR3 region was amplified using mCD16-specific VHH fragments as templates. In parallel, site-directed mutagenesis was performed on CDR1 and CDR2 using degenerate primers to generate mutant CDR1 (MCDR1) and mutant CDR2 (MCDR2). All three diversity elements were subsequently assembled stepwise with IGKV3-20 framework elements following the same procedure as previously described, generating the final VHHL fragments. All primers used in the assembly process are listed in Supplementary Table S6. The VHHL fragments were digested with SfiI (New England Biolabs, Ipswich, MA, USA) and cloned into phagemid pComb3x as previously described. The recombinant vector was electro-transformed into competent TG1 bacteria (Lucigen, Middleton, WI, USA) at 1.8 kV, 25 μF, and 200 Ω. Prewarmed 2× YT medium was added and incubated at 37°C with shaking at 225 rpm for 1 h. About 50 μl of the culture was 10-fold serially diluted in 500 μl of 2× YT medium and plated on 2× YT agar plates containing 2% glucose (w/v) and 100 μg/ml ampicillin. The plates were incubated overnight at 30°C, and the library diversity was calculated the next day by counting the number of colonies on agar plates. The remaining cultures were incubated for an additional 2 h at 37°C by adding 2% glucose (w/v) and 100 μg/ml ampicillin and infected with M13KO7 helper phages (Invitrogen, Waltham, MA, USA) when the optical density at 600 nm (OD600) reached 0.6. The cells were infected for 1 h at 37°C and harvested by centrifugation. The pellet was resuspended in 2× YT medium with ampicillin and kanamycin (100 μg/ml) and cultured overnight at 30°C for 14 h. The next day, the cells were centrifuged, and phages from the supernatant were precipitated by adding 20% (w/v) polyethylene glycol (PEG) 8000-NaCl (20% PEG8000, 2.5 mol/l NaCl) for 1 h at 4°C. Precipitated phages were collected by centrifugation, resuspended in sterile PBS, and frozen in aliquots of 10^13^ phages per tube at −80°C.

### Identification of mCD16-specific VHHL by phage screening

Panning protocols were carried out as previously described [6]. Briefly, the phage library was precipitated using PEG8000-NaCl and resuspended in sterile PBS. A total of 5 μg of biotinylated mCD16 antigen (ACROBiosystems, Beijing, China) was added to the suspension along with 3% MPBS, and the mixture was incubated at room temperature (RT) for 30 min. Then, 20 μl of streptavidin-coated magnetic beads (ThermoFisher) was added, followed by incubation at RT for an additional 1.5 h. After incubation, antigen-binding phages were isolated using a magnetic stand and used to infect TG1 cells in the logarithmic growth phase. After infection, the cells were pelleted by centrifugation and resuspended in 2× YT medium supplemented with ampicillin and kanamycin, and cultured overnight at 30°C for 14 h. The next day, phages were precipitated using PEG8000-NaCl, and the concentration was determined. A total of 10^12^ phages were used for the next round of biopanning, and four rounds were performed in total. After four rounds of panning, the enrichment of mCD16-specific VHHL was evaluated by polyclonal phage enzyme-linked immunosorbent assay (ELISA). Since mCD16 is a mouse Fc receptor, it can bind to the Fc region of the mouse-derived horseradish peroxidase (HRP)-conjugated anti-M13 mAb. To account for this potential interference, two additional controls were included in the ELISA design: phages from round 0 (pre-panning) and binding to an unrelated antigen (SARS-CoV-RBD). The actual enrichment of specific antibodies in round *n* (*n* = 1, 2, 3, 4) was calculated using the following formula: OD_405_ Real = (OD_405_ of mCD16 in round *n* − OD_405_ of SARS-CoV-RBD in round *n*) − (OD_405_ of mCD16 in round 0 − OD_405_ of SARS-CoV-RBD in round 0). Based on the above results, the panning round with a higher OD_405_ Real value was selected for dilution plating. The next day, individual colonies were picked for monoclonal ELISA analysis, except those located in the bottom-right corner of each plate, which served as negative controls. As in the polyclonal ELISA, due to the potential cross-binding between mCD16 and the Fc region of the mouse-derived HRP-conjugated anti-M13 mAb, the actual OD_405_ value for each well was calculated by subtracting the OD_405_ of the blank control (bottom-right well) from that of the test well. Colonies from wells with higher adjusted OD_405_ values were collected for sequencing.

### Crystallization and data collection

Crystallization of the purified L6 antigen-binding fragment was carried out using the sitting-drop vapor-diffusion method at 16°C using 96-well drop culture plates from Fastalbio. For each trial, 0.3 μl of protein solution (10 mg/ml in 20 mM HEPES, pH 7.5, 150 mM NaCl) was mixed with 0.3 μl of reservoir solution and equilibrated against 50 μl of reservoir in the well. Initial screening employed the Index, Crystal Screen I & II and PEG/Ion kits (Hampton Research), which survey a broad range of precipitants, salts, and pH values. Microcrystalline precipitates first appeared after approximately 1 week under acidic conditions consisting of 100 mM sodium acetate (pH 4.6) and 2.0 M ammonium sulfate. To improve crystal size and quality, additive screening was performed alongside fine-grid optimization of pH and precipitant concentration. Additive Screen HT and Silver Bullets (Hampton Research) were used to test a panel of compounds at 10% (v/v) in the reservoir solution, while the pH was varied in steps of 0.1 units around pH 4.6 and the ammonium sulfate concentration adjusted in 0.1 M increments. Through systematic evaluation of drop morphology and diffraction quality tests, a cocktail of additives—0.25% (w/v) benzidine, 0.25% (w/v) nicotinamide, 0.25% (w/v) pyromellitic acid, 0.25% (w/v) sulfaguanidine, and 0.02 M HEPES sodium, pH 6.8—was found to dramatically improve crystal diffraction. Final crystallization trials were set up in 24-well plates with 0.8 μl protein mixed with 0.8 μl optimized reservoir solution, yielding well-formed crystals measuring approximately 100–200 μm along their longest axis after 1 week of incubation. Prior to data collection, crystals were transferred into cryoprotectant solutions containing the reservoir solution supplied with 2 M Lithium sulfate. Individual crystals were then harvested and gently blotted to remove excess liquid and flash-cooled in liquid nitrogen. X-ray diffraction data were collected at 100 K on beamline BL10U2 at the Shanghai Synchrotron Radiation Facility, using a monochromatic beam of 0.9792 Å wavelength. A total rotation range of 360° was scanned in 1° oscillation steps with an exposure time optimized to minimize radiation damage while maintaining sufficient signal. Raw data were auto-processed by Aquarium pipeline or indexing, integration, scaling, and merging, resulting in a final dataset with 3.05 Å resolution (Table 1) [19].

**Table 1:** Data collection and refinement statistics of L6.

**Data collection**	**PDB entry 9VUZ**
Wavelength (Å)	0.9792
Space group	*P 62 2 2*
**Cell dimensions**	
*a*, *b*, *c* (Å)	90.9, 90.9, 165.7
α, β, γ (°)	90, 90, 120
Resolution (Å)	165.73–3.05 (3.13–3.05)^a^
*R*_merge_ (%)	20.8 (230)
*R*_pim_ (%)	4.7 (51.7)
*I/σ* (*I*)	12.1 (2.5)
Completeness (%)	100 (100.0)
Multiplicity	36.1 (38.4)
**Refinement**	
Resolution (Å)	15.31–3.05 (3.16–3.05)
No. reflections	8157 (792)
*R*_work_ */R*_free_ (%)	28.7/34.7
Number of non-hydrogen atoms	1780
Macromolecules	1780
Ligands	0
Average B-factor (Å^2^)	90.57
Macromolecules	90.57
Ligands	0
**R.m.s. deviations**	
Bond lengths (Å)	0.012
Bond angles (°)	1.85
**Ramachandran** ^**b**^	
Favored (%)	92.5
Allowed (%)	7.5
Outliers (%)	0

^a^Values in the parentheses refer to the highest resolution shell.

^b^As calculated by the Molprobity validation server.

### Structure determination and refinement

Molecular replacement was performed using PHENIX.phaser with an AlphaFold3-predicted model of L6, after trimming flexible loop regions to reduce model bias. Two copies of the predicted structure were positioned in the asymmetric unit, and initial phases were obtained with clear electron density for FRs. Initial model refining was performed manually in crystallographic object-oriented toolkit (COOT)—adjusting side-chain conformations, inserting water molecules where positive peaks appeared in 2Fo–Fc maps, and correcting register errors. Refinement in PHENIX.refine employed maximum-likelihood targets with individual atomic B-factor refinement and grouped Translation, Libration, and Screw-rotation (TLS) parameters. Refinement progressed until *R*_work_ and *R*_free_ converged, with final values indicating agreement between observed and calculated structure factors. The final model was validated using MolProbity, which confirmed that the vast majority of residues occupied favored regions of the Ramachandran plot and that geometry statistics met high-quality standards. Detailed validation metrics—including bond length and angle deviations, rotamer outliers, and clash scores—are provided alongside refinement statistics in Table 1.

### Structural analysis

Structural analysis was carried out using the Molecular Operating Environment (MOE) software package. The refined L6 structure was superposed onto the native human IGKV3-20 framework [Protein Data Bank (PDB): 5IBT] via Cα atom alignment of FRs to calculate root-mean-square deviation (RMSD). Interface analysis and identification of novel contacts were conducted using MOE’s contact analysis tools, which detected hydrogen bonds and hydrophobic interactions involving CDR residues. Surface area calculations quantified the buried interface between heavy and light chains, and the identified interactions were mapped and visualized. Structural figures, including ribbon diagrams, surface representations, and interaction maps, were rendered with MOE 2024. Electron density fit-to-model maps were generated and visualized in UCSF ChimeraX 1.9, ensuring clear presentation of density agreement and model accuracy for publication-quality figures.

### Profiling of the human immunoglobulin heavy chain subfamilies naturally paired with IGKV3-20

The analysis of immunoglobulin heavy chain variable (IGHV) subfamilies naturally paired with the IGKV3-20 light chain was performed based on antibody structures available in the PDB. First, antibody structures with ≥95% sequence identity to IGKV3-20 were retrieved using the advanced search function on the PDB website. Antibodies in scFv or Fab format were manually selected. FASTA sequence files were downloaded, and non-human sequences were excluded. Human heavy chain variable region sequences paired with the IGKV3-20 light chain were extracted and analyzed using IMGT/V-QUEST (https://www.imgt.org/IMGT_vquest/input) to identify their IGHV subfamilies. The output results were processed in Microsoft Excel and visualized using GraphPad Prism version 9.5.1.

### Construction, expression, and purification of novel IgG-format bsAbs

For the construction of IgG-format bsAbs based on modular assembly, the three CDRs of an anti-human HER2 (hHER2) VHH named 11A4 were grafted into the top five human IGHV subfamilies naturally paired with IGKV3-20 for humanization [20]. The humanized 11A4 (h11A4) fragment was recombined with the human constant region 1 (CH1) and IgG4 Fc fragment into the eukaryotic expression vector pTT5 to generate the heavy chain plasmid. For the construction of IgG-format bsAbs based on modular replacement, the original VH and CH1 regions of the parental mAbs were retained and separately recombined with either the human IgG1 or IgG4 Fc fragment into the pTT5 vector to generate the respective heavy chain plasmids. Primers were designed to separately amplify the VHHL fragment (L6 or L15) and the light chain constant region (CL) fragment, which were then recombined with the pTT5 to generate the light chain plasmids. Each IgG-format bsAb was expressed in 100 ml cultures. Briefly, Expi293F cells were seeded into sterile shaking flasks and cultured to reach a density of ~1.5 × 10^6^ cells/ml. Equal amounts of heavy and light chain plasmids (80 μg each) were added in 5 ml of Expi293F expression medium and filtered through a 0.22 μm membrane into expression medium containing 250 μl of PEImax 40 K and incubated for 20 min at RT. After incubation, the plasmid–PEImax mixture was added to the cell culture flask with continuous gentle shaking to ensure thorough mixing. Transfected cells were cultured in a shaker incubator for 5 days. After 5 days, the supernatants were collected and incubated overnight with Protein A resin (GenScript, Nanjing, China). The resin was loaded onto a chromatography column, washed with PBS (pH 7.4), and the IgG-format bsAbs were eluted with 0.1 M glycine (pH 3.0). The eluates were immediately neutralized with 1 M Tris-HCl (pH 9.0), followed by buffer exchange to PBS (pH 7.4) for subsequent analysis.

### Biolayer interferometry binding assays

For evaluating the specificity of different VHHLs, the biolayer interferometry (BLI) binding assays were performed on an Octet-RED96 (ForteBio, San Jose, CA, USA). Briefly, mCD16, biotinylated hCD3ε & CD3δ or biotinylated hCD16 at 5 μg/ml was separately loaded onto the amine-reactive second-generation or streptavidin-coated (SA) biosensors, then incubated with 2 μM VHHL for 300 s for association, and then immersed in 0.02% phosphate-buffered saline with Tween-20 (PBST) for another 300 s at 37°C for dissociation. For determining the binding kinetics of mCD16-specific VHHLs to mCD16 and Protein L, the his-tagged mCD16 at 10 μg/ml and biotinylated Protein L at 5 μg/ml were separately loaded onto the Ni-NTA and SA biosensors, followed by incubating with three-fold or two-fold serially diluted VHHLs starting at 2 μM in 0.02% PBST for 300 s for association, and then immersed in 0.02% PBST for another 300 s at 37°C for dissociation. Apart from differences in initial antibody concentrations, the binding kinetics for different isotype control mAbs against mCD16-His and novel IgG-format bsAbs against mCD16-His, Rabies virus (RABV)-His, and human tumor necrosis factor alpha (hTNF-α)-His were determined using the same procedures described previously. All curves were fitted by a 1:1 binding model using the Data Analysis software 11.1. All *K*_D_ values were determined with *R*^2^ values of greater than 97% confidence level.

### Enzyme-linked immunosorbent assay

After immunization, the serum antibody titers were measured by ELISA to assess the mCD16-specific immune response. Briefly, mCD16 antigen was coated onto 96-well microplates at 100 ng per well and incubated overnight at 4°C. The next day, the plates were washed three times with 0.05% PBST and then blocked with 100 μl of 3% MPBS per well at 37°C for 1 h. Alpaca serum collected post-immunization was thawed from −80°C. Once fully thawed, the serum was initially diluted 1:2000, followed by a series of two-fold serial dilutions. Subsequently, 50 μl of each dilution was added to the plates and incubated at 37°C for 1.5 h. After incubation, plates were washed three times with PBST. Then, 50 μl of HRP-Goat anti-Alpaca IgG (VHH domain) secondary antibody per well was added and incubated at 37°C for 45 min. After five additional washes with PBST, 50 μl of ABTS substrate (Invitrogen) was added to each well. The plates were incubated at 37°C for 10–30 min, and absorbance was measured at 405 nm. To evaluate the binding ability of different IgG-format bsAbs to hHER2 antigen, the same experimental protocol as mentioned above was adopted. Briefly, the human HER2-Fc antigen was coated in 96-well microplates, then blocked with 3% MPBS and added serially diluted antibodies. The HRP-conjugated anti-Fab (Sigma-Aldrich) secondary antibody was used, followed by adding ABTS substrate and detected at 405 nm.

### Size-exclusion high-performance liquid chromatography

The homogeneity of different VHHLs and IgG-format bsAbs was assessed using an Agilent 1260 Infinity II high-performance liquid chromatography system (Agilent Technologies, Santa Clara, CA, USA). Briefly, PBS filtered through a 0.22 μm membrane was used to flush the high-performance liquid chromatography (HPLC) system. Meanwhile, antibody samples were removed from −80°C and thawed on ice. Once thawed, the samples were centrifuged at 13 000× g for 15 min at 4°C. Then, 30 μl of the supernatant was transferred to sample vials for injection. Upon completion, absorbance at 280 nm over time was recorded using the built-in software. The homogeneity of the single-domain antibody samples was determined by calculating the area under the curve of the chromatographic profile.

### Flow cytometry

The binding of IgG-format bispecific antibodies to cell-surface hHER2 was evaluated by flow cytometry (FCM). Firstly, SKOV-3 cells with high expression of hHER2 were digested with trypsin, collected by centrifugation, and resuspended in sterile DPBS for counting. Cells were adjusted to the appropriate concentration and mixed at a 1:1 ratio with pre-diluted bsAbs or control mAbs (2×), followed by incubation at 4°C for 1.5 h. After incubation, the cells were washed three times with PBS and then incubated with Alexa Fluor 647-conjugated anti-human IgG (H + L) secondary antibody (Invitrogen) for 30 min at 4°C in the dark, followed by another three times of washing with PBS. The cells were resuspended in PBS and analyzed using an LSR Fortessa flow cytometer (BD Biosciences, San Jose, CA, USA). Data analysis and histogram generation were performed using FlowJo_v10.8 software.

### Statistical analyses

Statistical analyses were conducted using Prism software (Version 9.5.1, San Diego, CA, USA). Data were presented as mean ± SD from three independent experiments. Individual or multiple group comparisons were performed by the two-tailed unpaired Student’s t test. A statistically significant difference was defined as *P* < .05.

## Results

### Establishment of an engineering strategy for human light chain variable region

A well-behaved scaffold is essential for the efficient expression of light-chain single-domain antibodies. According to previous studies, the IGKV gene locus exhibits significantly less polymorphism than the IGHV locus [21–23]. Among all kappa light chain subfamilies expressed in adults and neonates, the IGKV3-20*01 allele has been reported to have a relatively high usage frequency [21, 24, 25]. Furthermore, the IGKV3-20 subfamily has been reported to be efficiently expressed in bacterial prokaryotic expression systems and can be successfully displayed on filamentous phages [26]. Based on these findings, IGKV3-20 was selected as the scaffold for the light-chain single-domain antibody in this study.

**Figure 1:**
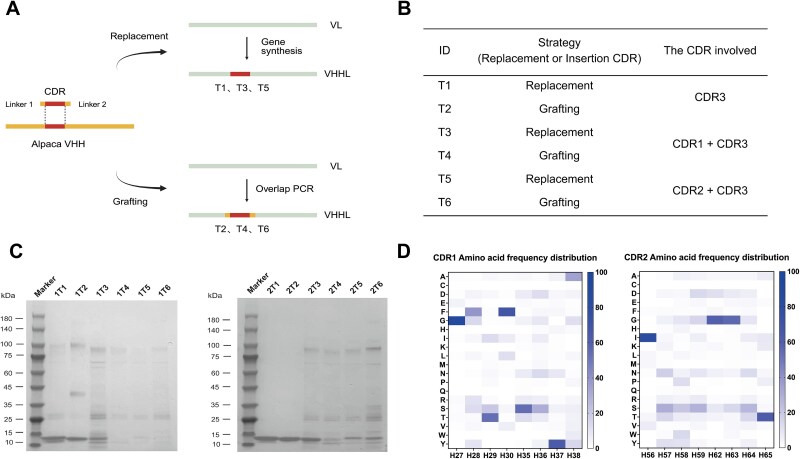
Establishment of a novel light-chain single-domain antibody engineering strategy. (A) Schematic illustration of two CDR transplantation strategies. The replacement-based strategy involves direct substitution of the original light-chain CDRs with alpaca-derived CDR sequences. In contrast, the grafting-based strategy introduces alpaca-derived CDRs along with their junctional framework sequences into the corresponding positions within the IGKV3-20 scaffold. (B) Summary table of alpaca heavy chain CDR transplantation strategies. (C) SDS-PAGE analysis of VHHL expression under different CDR transplantation strategies. The CDRs of two candidates were transplanted into the IGKV3-20 scaffold through “replacement” or “grafting,” respectively. Lanes labeled 1T1–1T6 and 2T1–2T6 represent VHHLs generated under different transplantation conditions. (D) Frequency distribution of amino acids in the heavy chain CDR1 and CDR2 regions. Residue positions on the horizontal axis are numbered according to the IMGT scheme, while the vertical axis shows the one-letter abbreviations for the 20 standard amino acids.

To generate light-chain single-domain antibodies with antigen-binding capability, we chose to graft heavy chain CDRs onto the IGKV3-20 scaffold due to the limited diversity of light chain CDRs. Previous studies have shown that camelid nanobody CDRs are longer than those of human VHs, which contributes to their higher sequence diversity [27–29]. In addition, immunization of alpacas can further enhance the likelihood of obtaining high-affinity binders. Therefore, we grafted the CDRs derived from a camelid immune library onto the IGKV3-20 scaffold to construct the recombinant light-chain single-domain antibodies (VHHLs). To identify a strategy that balances structural integrity with expression yield, we designed a series of engineering strategies (T1-T6) to optimize the CDR transplantation methods (Fig. 1A and B). Firstly, to reduce the potential structural disturbance caused by CDR transplantation, the number of grafted heavy chain CDRs was restricted to one or two. Secondly, given the critical role of CDR3 in antigen recognition and the high similarity of the junctional framework sequences of the CDR3 between alpaca IGHV and human IGKV3-20, the transplantation of CDR3 was included in all design strategies. Additionally, to minimize the potential impact of different junctional framework sequences between heavy and light chain CDR1/CDR2 regions on VHHL expression, two distinct CDR transplantation methodologies (termed “grafting” and “replacement”) were introduced. In “grafting,” the CDR together with junctional framework sequences of alpaca VHHs are both inserted into the IGKV3-20 by overlap extension PCR, which may have a great impact on the structural integrity of the light chain scaffold. In “replacement,” the light chain CDRs are fully swapped out for the alpaca CDRs while preserving the original human-derived junctional framework residues, thereby minimizing local disturbance. Based on the above guidance, two candidates were selected to evaluate the expression of VHHLs under different CDR transplantation strategies. The results showed that VHHLs exhibited better expression when only CDR3 was transplanted (T1 and T2), with a single, well-defined target band observed at ~ 15 kDa by SDS-PAGE (Fig. 1C). Moreover, we observed that the expression of the target protein was superior under T3 compared to T4, suggesting that the “replacement” of CDR imposes less structural disruption to the light chain scaffold than “grafting.” Therefore, we chose to transplant CDR1 and CDR2 into the IGKV3-20 scaffold using the “replacement” strategy. However, since the “replacement” is limited to substituting only fixed CDR sequences and does not allow for amplification of a complete CDR repertoire as the “grafting” strategy does, we sought to enhance the diversity of the heavy chain CDR1 and CDR2 through site-directed mutagenesis. Using the IMGT and AbYsis databases, we observed a high degree of similarity in amino acid preferences at corresponding CDR positions between alpaca and human. Therefore, we analyzed the amino acid frequency distributions of CDR1 and CDR2 from both alpaca and human heavy chains and designed two distinct mutagenesis strategies based on the selected positions. (Fig. 1D, Supplementary Tables S4 and S5). In summary, we established a light chain engineering approach that combines CDR3-grafting with site-directed mutagenesis of CDR1 and CDR2, providing a rational foundation for the subsequent development of VHHLs with antigen-binding ability.

### Identification of mCD16-specific VHHLs

**Figure 2:**
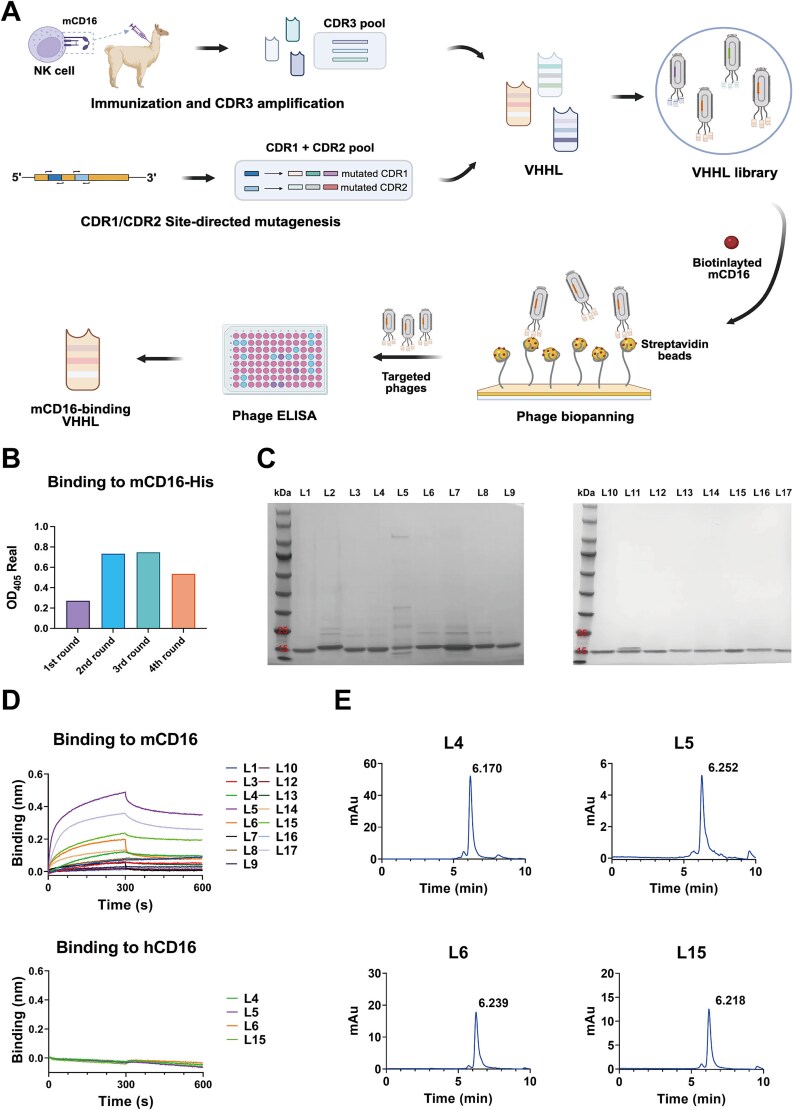
Identification of novel light-chain single-domain antibodies against mCD16 by phage panning. (A) Schematic overview of the construction and biopanning workflow of the VHHL phage display library. (B) Phage enrichment across four rounds of biopanning, as determined by polyclonal phage ELISA. (C) SDS-PAGE analysis of the purity of candidate VHHLs. The leftmost lane in each gel represents the protein marker. L1–L17 indicate the corresponding names of each VHHL. (D) Antigen-binding specificity of VHHLs evaluated by BLI. All VHHLs were tested at a concentration of 2 μM. (E) SEC-HPLC analysis of the homogeneity of mCD16-specific VHHLs. The horizontal axis indicates retention time, and the vertical axis represents absorbance at 280 nm.

To validate the human light chain engineering strategy established above, mouse CD16 (mCD16) was selected as the target antigen. mCD16 is widely expressed on immune cells, including natural killer cells, neutrophils, polymorphonuclear leukocytes, monocytes, and macrophages and serves as a critical target in bsAb [30]. To obtain VHHLs with mCD16-binding ability, an alpaca was immunized with mCD16 (Supplementary Fig. S1A). After four rounds of immunization, serum ELISA confirmed the successful generation of mCD16-specific antibodies in the alpaca (Supplementary Fig. S1B). Subsequently, the heavy chain variable regions were amplified from alpaca PBMCs using a two-step PCR approach (Supplementary Fig. S1C). Following the VHHL engineering strategy, individual diversity elements were amplified and assembled with the light chain scaffold through stepwise assembly, ultimately resulting in the successful amplification of VHHL fragments (Fig. 2A). Two VHHL phage libraries were constructed and pooled for four rounds of biopanning. Polyclonal phage ELISA results demonstrated a significant enrichment of mCD16-specific binding phages over successive rounds of selection (Fig. 2B) and a panel of 17 candidates was selected by monoclonal ELISA for further validation. All candidate VHHLs were successfully expressed, with most exhibiting good purity on SDS-PAGE (Fig. 2C). To further evaluate the antigen-binding specificity of the candidate VHHLs, binding assays were conducted using BLI (L2 and L11 were excluded from the analysis due to abnormal SDS-PAGE band patterns). The results demonstrated that four mCD16-specific VHHLs were identified. They bound to mCD16 and showed no detectable binding to human CD3 or CD16 protein (Fig. 2D, Supplementary Fig. S2).

To further evaluate the biophysical characteristics of the mCD16-specific VHHLs, size-exclusion (SEC)-HPLC was used to examine the homogeneity of each candidate. The results showed that all VHHLs exhibited good homogeneity with no apparent aggregation (Fig. 2E). Among them, L6 and L15 exhibited more favorable profiles, with single, sharp peaks. Therefore, these two VHHLs were selected for further validation.

### Binding activity of mCD16-specific VHHLs

**Figure 3:**
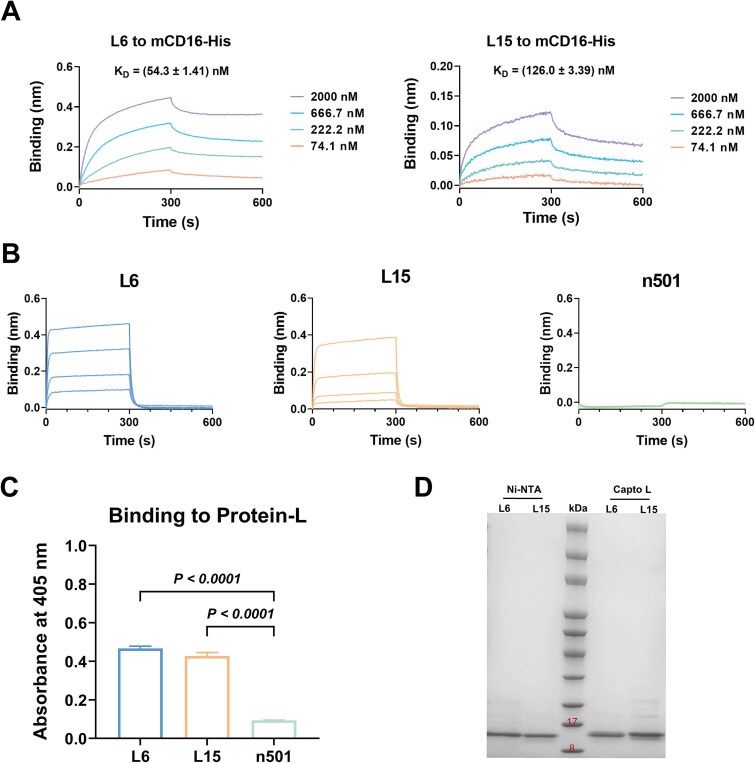
Characterization of the mCD16-specific light-chain single-domain antibodies L6 and L15. (A) Binding kinetics of L6 and L15 to mCD16 antigen as measured by BLI. (B) BLI assessment of L6 and L15 binding to biotinylated Protein L using SA biosensors. The starting concentration of each antibody was 2 μM, followed by a 2-fold serial dilution. The fully human sdAb n501, previously identified by our team, was included as a negative control. (C) Protein L binding of L6 and L15 by ELISA. All antibodies were tested at a concentration of 2 μM, and each measurement was performed in triplicate. (D) Purification of L6 and L15 using different resins. The central lane represents the protein marker. Lanes to the left and right show VHHL proteins purified using Ni-NTA resin and Capto L resin, respectively.

We next characterized the binding kinetics of L6 and L15 to mCD16 using BLI. The results showed that L6 bound to mCD16 with a relatively high affinity, with an equilibrium dissociation constant (*K*_D_) of 54.3 ± 1.41 nM. In comparison, L15 exhibited a *K*_D_ of 126.0 ± 3.39 nM (Fig. 3A).

According to previous studies, one intrinsic feature of human kappa light chain variable domains (subtypes 1, 3, and 4) is their ability to bind protein L [31, 32]. Given the potential structural disturbance of heavy chain CDR grafting on the light chain scaffold, we investigated whether this modification abrogated the protein L binding by assessing the interactions of L6 and L15 to protein L via ELISA and BLI. As expected, both VHHLs retained the ability to bind protein L (Fig. 3B and C). In contrast, n501—a fully human heavy chain single-domain antibody previously identified in our laboratory—showed no detectable binding to protein L [7]. Next, we attempted to purify L6 and L15 using Capto L resin. The results showed that both VHHLs were successfully purified, and the purity of the target proteins was comparable to that obtained by Ni-NTA resin (Fig. 3D). This indicates that L6 and L15 can be efficiently purified without the need for exogenous tags, thereby simplifying the purification process and significantly reducing production costs in industrial settings. Taken together, the above findings confirm that the selected mCD16-specific VHHLs possess favorable biophysical properties, highlighting the successful establishment of a robust light-chain single-domain antibody development platform.

### Crystallization and structural determination of light-chain single-domain antibody L6

**Figure 4:**
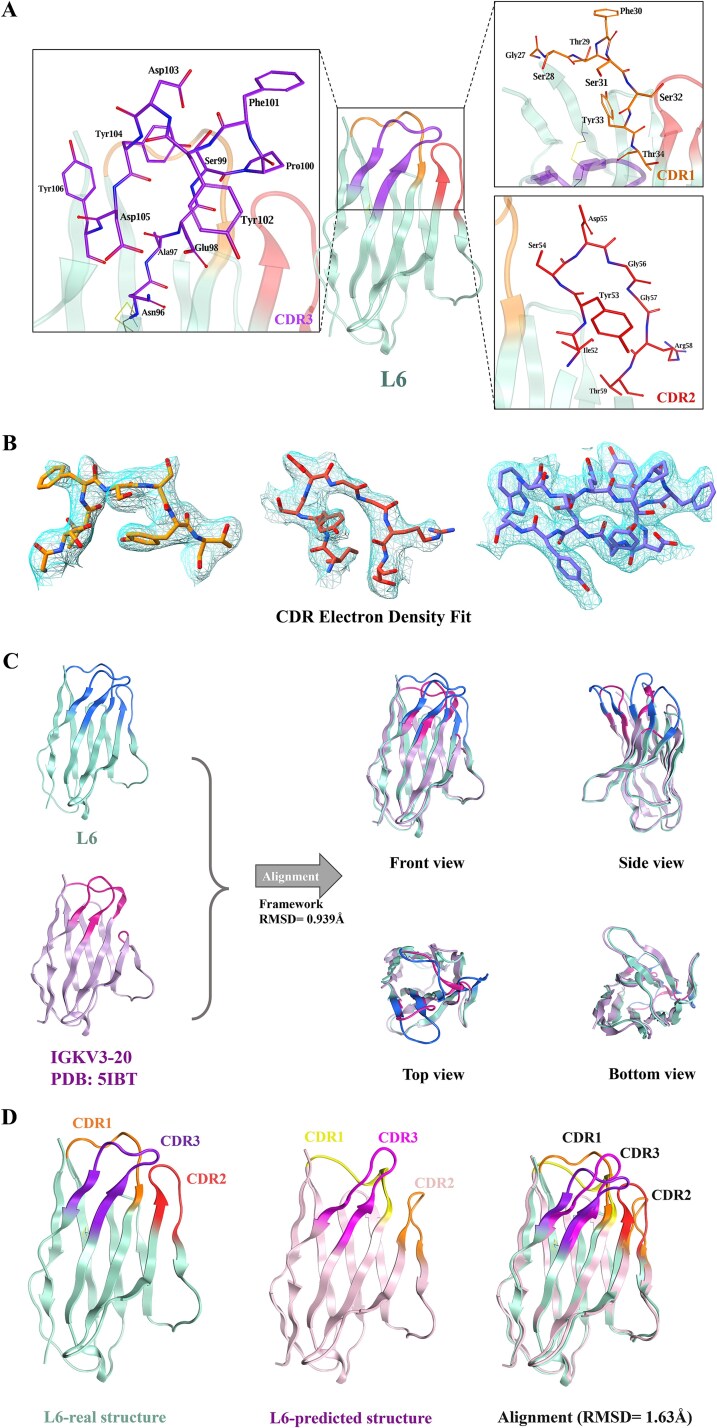
Crystal structure of L6 and comparison with AlphaFold3 predictions and IGKV3–20 framework. (A) Cartoon representation of L6, highlighting the β-sheet core and CDRs. (B) Electron density maps (2Fo–Fc, contoured at 1.0σ) overlaid on CDR loops, showing clear density, and excellent agreement with the refined model. CDR1, orange; CDR2, red; CDR3, purple. (C) Structural comparison with the native IGKV3-20 framework. Superposition of L6 and IGKV3-20 reveals minimal framework perturbation, with CDRs contrasting due to sequence modifications. (D) Superposition of the crystal structure and AlphaFold3 model of L6.

Light-chain single-domain antibody L6 was successfully crystallized under optimized conditions, yielding crystals with a resolution of 3.05 Å (Table 1, PDB: 9VUZ). The crystal structure of L6 was determined using molecular replacement with a template derived from AlphaFold3 predictions. The final refined model exhibited high stereochemical quality, with an *R*_work_/*R*_free_ of 27.7%/32.6% and a RMSD of 0.2 Å for bond lengths and 0.57° for bond angles. The structure revealed that L6 adopts a typical immunoglobulin variable domain fold, comprising seven β-strands arranged in a β-sheet core, with three CDRs extending outward to form an antigen-binding surface (Fig. 4A). Notably, the electron density maps showed clear and continuous density for all three CDR loops, indicating high confidence in loop placement and conformational assignment (Fig. 4B). This superior map-model agreement further supports the structural integrity and precision of the refined L6 model.

To evaluate the structural impact of CDR grafting, the experimentally determined structure of L6 was superimposed onto its native light chain framework, IGKV3-20 (PDB: 5IBT). Structural alignment was performed using MOE's structure alignment module based on Cα atoms of the FRs. The resulting RMSD of 0.853 Å indicates minimal backbone perturbation despite extensive CDR sequence modifications (Fig. 4C). Notably, the grafted CDRs in L6 adopt a more compact, hook-like conformation compared to the more dispersed CDR loops in the IGKV3-20 template. This rearrangement likely enhances antigen contact by concentrating binding residues in a co-planar orientation. The analysis suggests that the IGKV3-20 scaffold is structurally tolerant to CDR length and sequence variation, highlighting its suitability as a stable framework for light chain antibody engineering. The crystal structure of L6 adopts a canonical immunoglobulin fold composed of nine antiparallel β-strands, with CDR1, CDR2, and CDR3 forming a hook-like architecture projecting from the β-sandwich core (Fig. 4A). These loops—colored orange, red, and purple—extend co-planarly, forming a contiguous and accessible antigen-binding surface. When compared to AlphaFold3 predictions, significant conformational discrepancies were observed (Fig. 4D). CDR1 in the experimental structure protrudes laterally, while the predicted model presents a more medially shifted loop (yellow). CDR2 showed overall backbone agreement (Cα RMSD: 0.89 Å), though substantial variation in side-chain rotamers was detected (χ1 angle deviation >40°). CDR3 demonstrated the most notable difference with the predicted loop extending vertically, disrupting the planar antigen-binding surface observed in the experimental model (magenta vs. purple). Collectively, the global RMSD between the crystal structure and the AlphaFold3 model was 1.63 Å, with 78% of the deviation localized to the CDRs, underscoring the limitations of current predictive algorithms in modeling engineered antibody topologies.

### Development of a novel IgG-format bsAb platform based on VHHL

**Figure 5:**
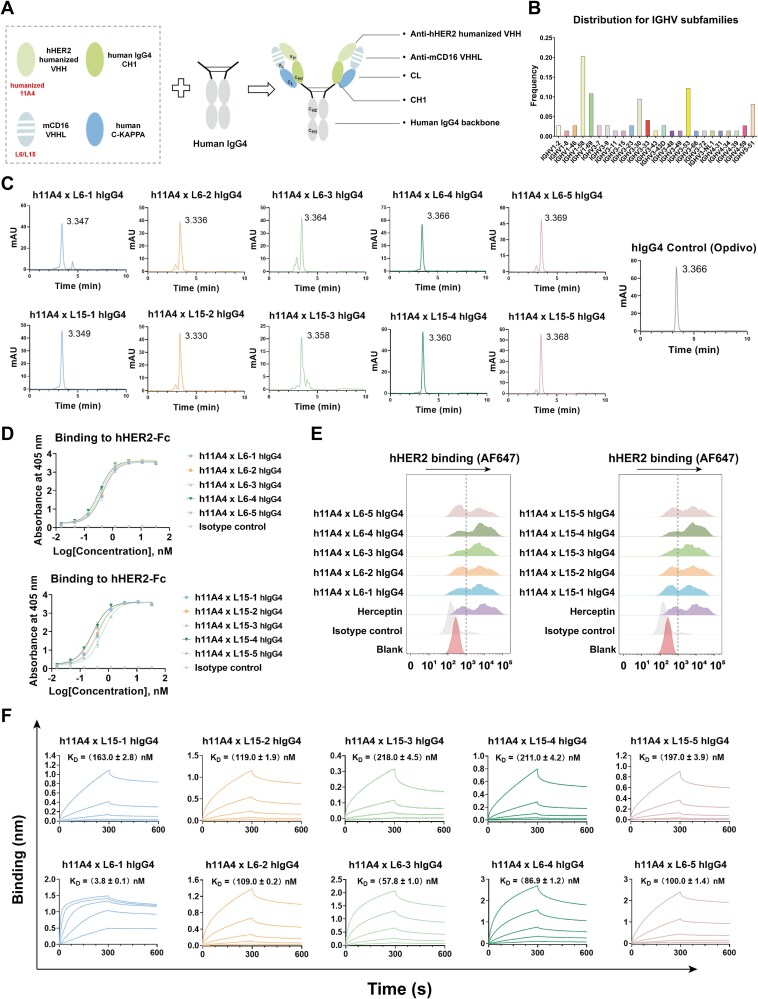
Design and characterization of novel IgG-format bispecific antibodies based on modular assembly. (A) Schematic illustration of the novel IgG-format bsAb based on a modular assembly strategy. (B) Frequency distribution of human IGHV subfamilies naturally paired with IGKV3-20. (C) SEC-HPLC evaluation of the homogeneity of each hIgG4 bsAb. (D) Assessment of the binding of each hIgG4 bsAb to hHER2-Fc antigen, as determined by ELISA. Antibodies were tested starting at 33 nM with a three-fold serial dilution. Data are presented as mean ± SD from three biological replicates. (E) Flow cytometry analysis of the binding of each hIgG4 bsAb to SKOV-3 cells. Antibody concentration was set at 1 μM. Anti-hHER2 mAb Herceptin was used as a positive control. (F) Binding kinetics of each hIgG4 bsAb to mCD16 antigen as determined by BLI. The starting concentration for each bsAb was 1 μM, followed by a three-fold serial dilution. In all panels, hIgG4 bsAbs labeled 1 to 5 utilize heavy chain IGHV subfamilies IGHV1-58, IGHV1-69, IGHV3-30, IGHV3-53, and IGHV5-51, respectively. A commercial hIgG4 mAb, Opdivo, was included as an isotype control.

Next, we attempted to reassemble the VHHL into a full-length IgG backbone, aiming to construct a novel format of bsAb that is structurally identical to conventional IgG. To minimize interference between chains that could potentially compromise binding, we initially selected camelid nanobodies as the heavy chain component of the novel IgG-format bsAb, owing to their relatively independent antigen-binding capacity (Fig. 5A). In this modularly assembled bsAb, a previously reported anti-human HER2 nanobody 11A4 was selected as the variable region of the heavy chain, and incorporated the mCD16-specific VHHL domain L6 or L15 as the light chain. Since camelid-derived IGHVs are incompatible with the human light chain backbones, it was necessary to identify human IGHV subfamilies that are capable of pairing with the human IGKV3-20 light chain scaffold. To this end, we analyzed all antibodies in the PDB database that contain IGKV3-20 as their light chain and compiled the frequency distribution of the corresponding IGHV subfamilies (Fig. 5B). As shown in the figure, IGHV1-58 was the most frequently paired subfamily, accounting for approximately one-fifth of all sequences. Additionally, IGHV1-69, IGHV3-30, IGHV3-53, and IGHV5-51 also showed relatively high pairing frequencies with IGKV3-20. Based on this analysis, these five IGHV subfamilies were selected as candidate scaffolds for the humanization of 11A4. Since mCD16 may cross-react with the Fc region of human IgG (hIgG), it was essential to eliminate potential Fc-mediated binding to mCD16 when designing IgG-format bsAbs. According to previous reports, the interaction between mCD16 and human IgG4 (hIgG4) is relatively weak [33]. To validate this, we evaluated the binding affinities of control mAbs bearing different human IgG subclasses (hIgG1, hIgG2, and hIgG4) to mCD16 using BLI (Supplementary Fig. S3). Consistent with previous reports, the results showed that the hIgG1 isotype exhibited the strongest binding to mCD16, whereas hIgG2 and hIgG4 displayed substantially weaker interactions. Based on these findings, we chose hIgG4 for constructing the bsAb to minimize Fc-mediated cross-reactivity against mCD16 (Fig. 5A).

Next, we constructed and successfully expressed all hIgG4 format bsAbs. The SEC-HPLC analysis demonstrated good homogeneity for the majority of the hIgG4 format bsAbs, characterized by a distinct main peak (Fig. 5C). Notably, all tested antibodies exhibited a retention time of approximately 3.36 min, closely matching that of the hIgG4 control mAb nivolumab (Opdivo). This indicates that the VHHL-based IgG-format bsAbs exhibit high structural integrity compared to conventional mAbs. Next, to examine whether the heavy and light chains of the IgG-format bsAbs retained their original antigen-binding abilities after assembly, we evaluated their binding to respective target antigens. Firstly, ELISA was performed to assess the heavy chain binding activity, and all hIgG4 format bsAbs demonstrated specific binding to hHER2 (Fig. 5D). In a cellular context, flow cytometry analysis further confirmed their binding to hHER2-positive SKOV-3 cells, with patterns comparable to the positive control trastuzumab (Herceptin), whereas the isotype control Opdivo showed no detectable binding (Fig. 5E). In parallel, to further evaluate the antigen-binding of the light chains in all bsAbs. BLI was performed and revealed that the VHHL-derived light chains in all IgG-format bsAbs maintained their binding capacity to mCD16 (Fig. 5F). Together, these findings validate the feasibility of incorporating VHHLs into an IgG backbone using the modular assembly strategy.

**Figure 6:**
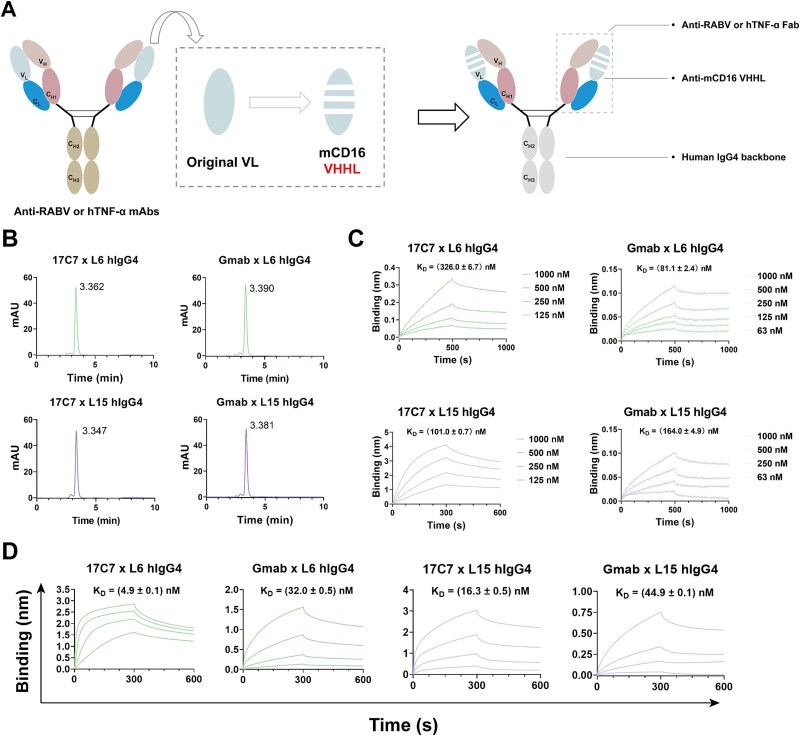
Design and characterization of novel IgG-format bispecific antibodies based on modular replacement. (A) Schematic representation of the IgG-format bsAb design based on a modular replacement strategy. (B) Evaluation of the homogeneity of each hIgG4 bsAb by SEC-HPLC. (C) Binding kinetics of the hIgG4 bsAbs to RABV and hTNF-a, as measured by BLI. The starting concentration for each bsAb was 1 μM with a two-fold serial dilution. (D) BLI analysis of the binding kinetics between each hIgG4 bsAb and mCD16. The starting concentration for each bsAb was 333 nM with a three-fold serial dilution.

The above results inspired us with a further design strategy: whether the light chain of a mAb could be directly replaced with a VHHL domain to endow it with additional antigen-binding specificity. By structural screening of antibody–antigen complexes from the PDB database, 17C7 (targeting RABV), and golimumab (Gmab, targeting hTNF-α) were selected as models for proof-of-concept (Supplementary Fig. S4, Supplementary Tables S7 and S8). Firstly, we constructed IgG1 format bsAbs by only replacing the native light chains with VHHLs, while maintaining the original hIgG1 framework of the parental mAb (Supplementary Fig. S5A). The resulting hIgG1 bsAbs generated through a modular replacement strategy were successfully expressed and exhibited molecular weights and SEC-HPLC retention times comparable to those of the conventional mAb CR3022, indicating preserved structural integrity (Supplementary Fig. S5B and C). Given that the hIgG1 Fc region exhibits cross-reactivity with mCD16, all bsAbs were further reconstructed into IgG4 format (Fig. 6A). All hIgG4 bsAbs were also efficiently expressed and exhibited structural profiles highly comparable to conventional mAbs, as confirmed by SEC-HPLC (Fig. 6B). BLI assays further demonstrated that both the substituted light chains and the original heavy chains retained their binding to corresponding antigens (Fig. 6C and D). Collectively, these results demonstrate the feasibility of reassembling VHHLs into conventional mAbs, thereby enabling the generation of a new class of bispecific antibodies that are structurally identical to native IgG, providing a novel strategy for the development of next-generation antibody therapeutics.

## Discussion

As one of the popular research subjects in the field of biopharmaceuticals in recent years, single-domain antibodies have attracted much attention due to their small molecular weight (about 12–15 kDa) and superior physicochemical properties [34]. Unlike conventional sdAbs derived from heavy chains, light-chain sdAbs represent a novel class of antibody domain that utilizes the variable region of the human light chain for antigen recognition. In the present study, we established a novel platform for the development of recombinant light-chain single-domain antibodies (VHHLs). To enhance the antigen-binding affinity of the VHHLs, we proposed an innovative strategy involving the grafting of heavy chain CDRs from a camelid-derived immune library into the human light chain scaffold IGKV3-20. This approach was based on the observation that the human heavy chain variable region subfamilies exhibit greater diversity compared to light chains [35]. Additionally, using immune-derived antibody fragments as diversity donors improved the likelihood of obtaining high-affinity binders.

To ensure the efficient and complete grafting of heavy chain CDRs into the light chain scaffold, it was necessary to introduce additional heavy chain junctional framework sequences into the primers. However, due to the low sequence homology between heavy and light chain scaffolds, directly replacing the corresponding light chain regions with heavy chain-derived framework sequences may adversely compromise the structural integrity of the light chain scaffold. Therefore, it was essential to explore suitable CDR grafting strategies to balance sequence diversity and structural stability in the engineered VHHLs. Through systematically evaluating the different CDR grafting strategies, we found that the additional heavy chain junctional framework sequences indeed impaired VHHL expression. Moreover, reducing the number of grafted CDRs led to improved expression levels of the target proteins. Based on these findings, we established a light chain engineering strategy that involves the amplification of CDR3 and the site-directed mutagenesis of CDR1 and CDR2. Building upon this strategy, we selected mCD16 as the target antigen to construct a VHHL phage display library. Following four rounds of biopanning, a panel of candidate molecules was successfully isolated. SDS-PAGE analysis confirmed high purity for all VHHL proteins. Subsequent SEC-HPLC and BLI assays identified two lead clones, L6 and L15, with favorable biophysical properties, including strong binding specificity, high homogeneity, and robust affinity toward mCD16. Interestingly, both L6 and L15 retained their capacity to bind Protein L despite the grafting of camelid CDRs, enabling one-step affinity purification via Capto L resin without the need for exogenous purification tags. This tag-free purification simplifies downstream processing and enhances production efficiency. Taken together, these findings highlight the feasibility of our light chain engineering strategy for the generation of well-behaved VHHLs.

The crystal structure of the engineered light-chain single-domain antibody L6 provides key insights into how the spatial arrangement of CDRs contributes to antigen recognition. Unlike conventional antibodies or camelid VHHs, which often present CDRs in a staggered or turret-like configuration, L6 exhibits a distinctive hook-shaped topology in which all three CDR loops project co-planarly from the β-sandwich framework. This geometry effectively creates a broad, contiguous paratope surface that may enhance the antibody’s capacity to engage with flat or groove-like epitopes, such as those found on the surface of membrane receptors like mCD16. The co-planar alignment may also facilitate simultaneous multi-residue contact across the epitope, improving binding affinity through increased interface area and reducing entropic penalties upon complex formation. Beyond its functional architecture, L6’s scaffold, which is based on the human IGKV3-20 scaffold, demonstrates exceptional structural robustness in tolerating CDR grafting. The low RMSD of 0.939 Å between L6 and the native IGKV3-20 structures indicates that the backbone geometry remains largely unperturbed despite extensive sequence alterations within the hypervariable loops. This structural tolerance supports the use of IGKV3-20 as a reliable scaffold for the engineering of human-derived sdAbs. Its compatibility with diverse CDR configurations also suggests potential for high-diversity sdAb library construction, especially in phage or yeast display systems. The stability of the IGKV3-20 in accommodating varied loop lengths and conformations further reinforces its promise as a modular platform for developing next-generation therapeutics. Interestingly, comparison with AlphaFold3 predictions revealed that pronounced discrepancies in CDR conformations of L6, despite overall preservation of the immunoglobulin fold. While the FRs were accurately modeled, the CDR loops—particularly CDR1 and CDR3—exhibited significant deviations in position and orientation. Moreover, the global RMSD between predicted and crystal structures of L6 reached 1.63 Å, with approximately 78% of the deviation arising from the CDRs. Notably, AlphaFold3 failed to predict the co-planar CDR arrangement observed experimentally, instead rendering a more staggered configuration that may underestimate paratope accessibility. These findings highlight current limitations in applying general-purpose protein structure prediction tools to engineered antibody domains, where loop grafting and non-natural topologies introduce additional modeling complexity. While AlphaFold3 offers a valuable starting point for model generation, our data underscore the necessity of experimental validation when precise loop positioning is critical, especially in applications involving epitope targeting or structure-based antibody design.

To successfully reconstruct VHHLs into full-length monoclonal antibodies and develop novel IgG-format bsAbs, two critical criteria must be fulfilled. First, the light and heavy chains of the bsAb must be capable of correctly pairing, as in native IgGs, to preserve structural integrity. Second, the individual antigen-binding functions of both chains must be preserved upon bsAb assembly. To achieve this, it is of great importance to identify human IGHV subfamilies that are naturally compatible with the IGKV3-20 scaffold. Statistical analysis of antibody structures in the PDB database revealed a set of human IGHV subfamilies that frequently pair with IGKV3-20. In parallel, to minimize potential interference of VHHLs on heavy chain functionality of the bsAb, we utilized camelid-derived nanobodies as the heavy chain component for their structural independence and ability to retain antigen-binding function when reformatted into distinct formats (such as nanobody-Fc fusion proteins). Guided by this rationale, we selected five IGHV subfamilies with the highest natural pairing frequencies with IGKV3-20 and grafted the anti-hHER2 nanobody 11A4 onto these human IGHV subfamilies to generate humanized VHHs. These humanized VHHs were then paired with VHHLs to construct bsAbs. Experimental validation showed that the resulting bsAbs exhibited structural and physicochemical properties comparable to traditional IgGs. Furthermore, binding assays demonstrated that both the VHHL-derived light chain and the nanobody-derived heavy chain retained their original antigen-binding specificity, confirming the functional integrity of the bispecific constructs. Encouraged by the above results, we sought to further broaden the applicability of the novel bsAb platform. Since the previously constructed bsAbs were assembled from distinct antibody modules, they did not fully recapitulate the architecture of native IgG molecules to some extent. To establish a more streamlined and generalizable strategy, we aimed to directly replace the light chains of conventional mAb with VHHLs, while retaining all other components unchanged. This approach would enable rapid and facile conversion of existing mAbs into bsAbs. To minimize the risk of completely disrupting antigen recognition by the heavy chain upon light chain substitution, we screened complex structures from the PDB database and selected two antibodies, 17C7 and Gmab, as candidates. As anticipated, all reconstructed IgG-format bsAbs were successfully expressed and preserved structural features consistent with native IgGs. Moreover, antigen-binding assays confirmed that both the VHHL-derived light chains and the original heavy chains maintained their respective antigen-binding specificities.

Production cost remains one of the critical considerations in antibody drug development [36]. Compared to conventional bsAbs, the novel IgG-format bsAb platform developed in this study offers substantial advancements across multiple dimensions, effectively establishing a “ready-to-use” bsAb platform. Structurally, this bsAb format is relatively simple, requiring only the replacement of the parental light chain with an antigen-binding VHHL. The transfection procedure is also identical to that of traditional mAbs, involving co-transfection of the VHHL light-chain plasmid with the original heavy-chain plasmid. Moreover, the bsAbs produced using this platform exhibited high expression yields, reaching tens of milligrams per liter, thereby significantly reducing manufacturing costs.

Nevertheless, the bsAb platform described in this study has certain limitations that require further improvement. One major challenge lies in the high cost associated with the selection of antigen-binding VHHL molecules. Generating such binders against specific target antigens typically necessitates alpaca immunization, which requires considerable time, labor, and financial resources. Therefore, this strategy is not suitable for the rapid generation of IgG-format bsAbs against diverse antigens in a short period. Additionally, although this study resolved the crystal structure of the light-chain single-domain antibody L6, the precise interaction interface between L6 and the mCD16 antigen remains unknown. Future work may involve structural characterization of the L6-mCD16 complex to gain deeper insight into the molecular basis of antigen recognition by light-chain single-domain antibodies.

In summary, this work presents the establishment of a VHHL-based antibody platform through rational antibody engineering. Using phage panning, we successfully obtained mCD16-specific VHHL molecules with favorable biophysical properties and resolved their crystal structure via X-ray crystallography. Further reconstitution of these VHHLs into full-length mAbs demonstrated a novel type of bsAb platform that structurally mimics natural IgG. This work offers a valuable reference for the future development of next-generation antibody therapeutics.

## Supplementary Material

Supplementary_materials_tbaf020

## Data Availability

All data in this study are available on reasonable request.

## Animal research statement

All experimental procedures involving animals were conducted in accordance with Institutional Animal Care guidelines approved under IACUC protocols at Nami Biotechnology (Shanxi, China).
