# Supplementary material for: Engineering a novel light-chain single-domain antibody to enable IgG-format bispecific antibody design
Source: Antib Ther. 2025 Oct 17;8(4):301–16. doi: 10.1093/abt/tbaf020 (PMC12598741; doi:10.1093/abt/tbaf020)
Supplement: Supplementary_materials_tbaf020 [file supplementary_materials_tbaf020.docx]

**Engineering a novel light-chain single-domain antibody to** **enable IgG-format bispecific antibody design**

Mingkai Wang^1,#^, Qingyuan Xu^1,2,#^, Yu Kong^1,3^, Yuxuan Zhong^1^, Feng Yin^1,2^, Litong Liu^1^, Zhenlin Yang^2,*^, Tianlei Ying^1,3,*^, Yanling Wu^1,3,*^

^1^Key Laboratory of Medical Molecular Virology (MOE/NHC/CAMS) and Shanghai Institute of Infectious Disease and Biosecurity, School of Basic Medical Sciences, Department of Pulmonary and Critical Care Medicine, Department of Liver Surgery and Transplantation, Zhongshan Hospital, Fudan University, Shanghai 200032, China

^2^Shanghai Key Laboratory of Lung Inflammation and Injury, Department of Pulmonary Medicine, Zhongshan Hospital, Fudan University, Shanghai 200032, China

^3^Shanghai Engineering Research Center for Synthetic Immunology, Shanghai 200032, China

#These authors contributed equally

*Corresponding authors: Yanling Wu. School of Basic Medicine, Fudan University, Shanghai 200032, China; E-mail: yanlingwu@fudan.edu.cn

**Supplementary materials**

**
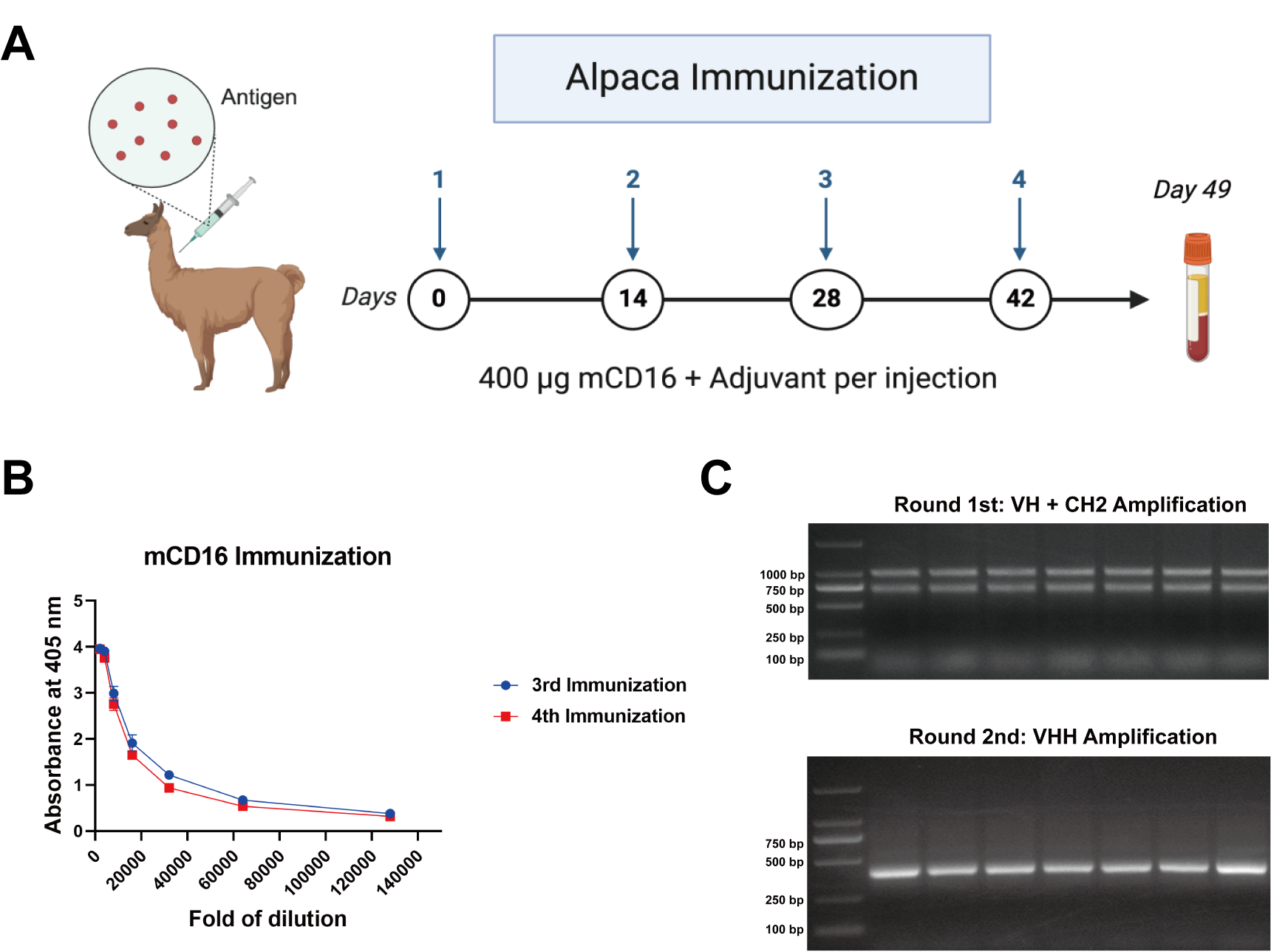
**

Supplementary Figure S1 Alpaca immunization and VHH amplification.

1. Schematic diagram of the alpaca immunization procedure.
   (B) Evaluation of serum antibody titers following alpaca immunization by ELISA. The horizontal axis represents the dilution ratio, and the vertical axis indicates the binding activity to the mCD16 antigen.
   (C) Amplification of the alpaca VHH fragments using a two-step PCR strategy.


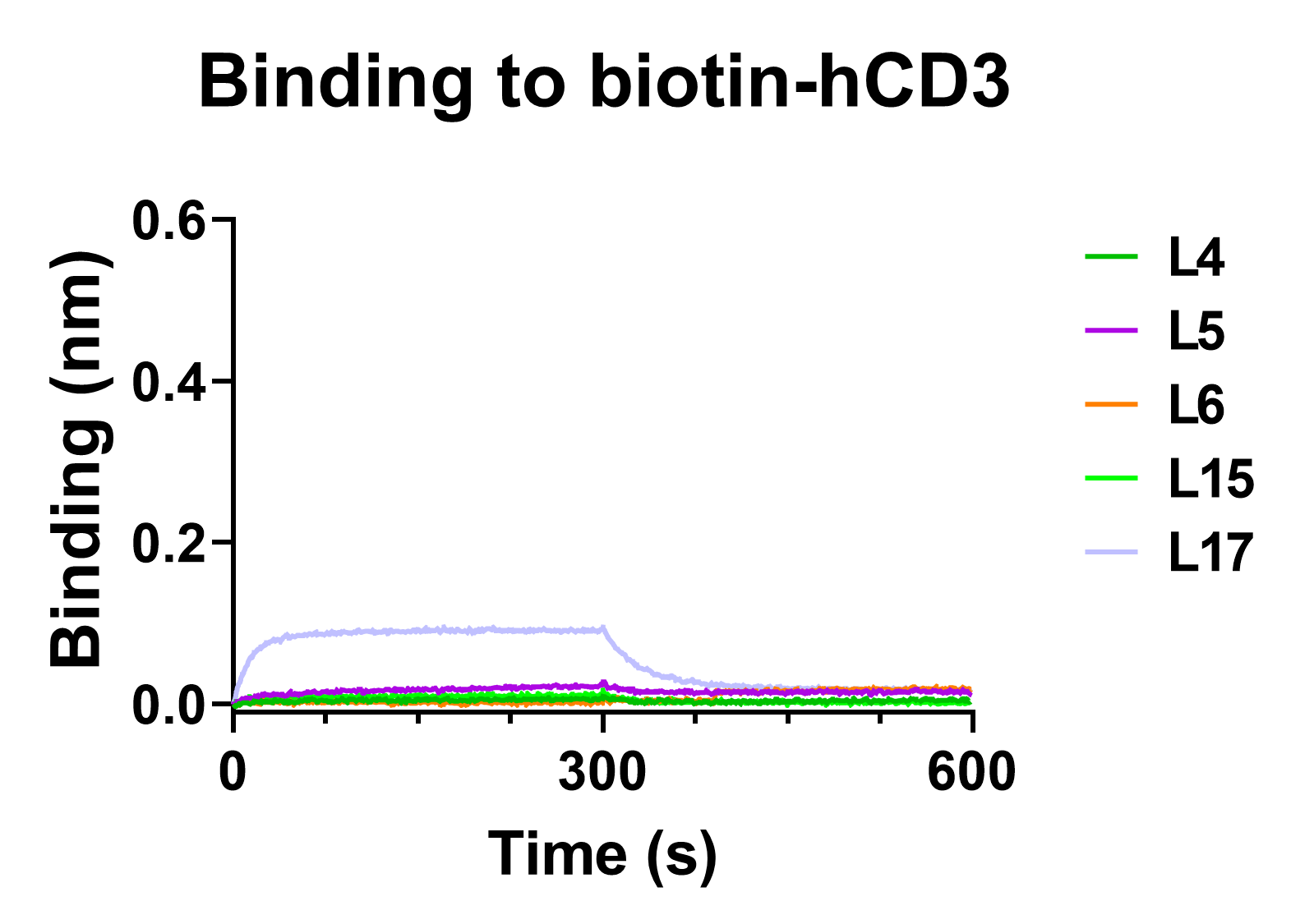


**Supplementary Figure S2 Non-specific binding of VHHL to unrelated antigens assessed by BLI.**

Five mCD16-binding VHHLs were tested for non-specific binding to human hCD3ε & CD3δ . All VHHLs were tested at a concentration of 2 µM.

**
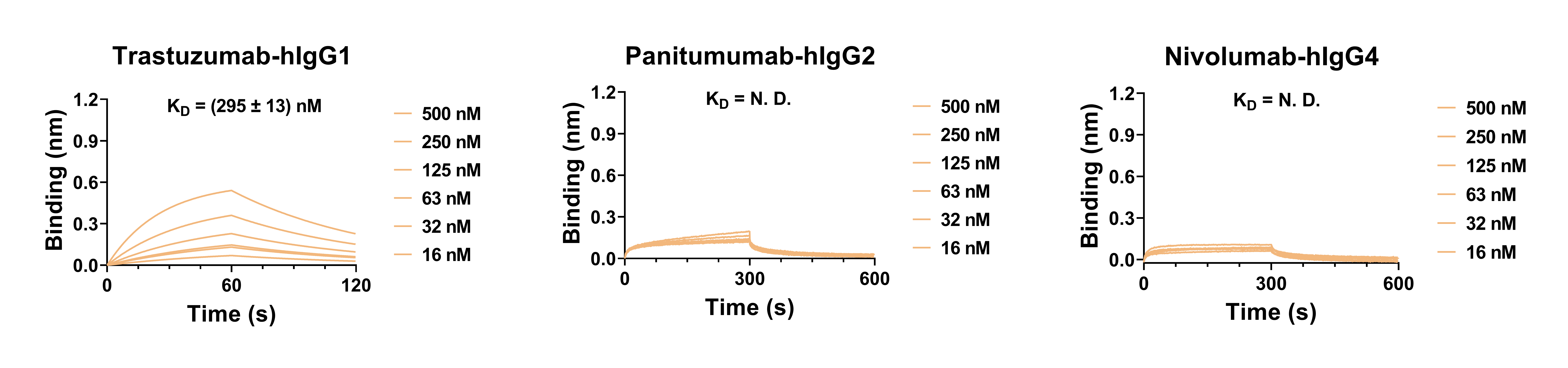
**

**Supplementary** Figure S3 Characterization of mCD16 cross-reactivity among different human IgG subclasses by BLI.

Use commercial mAbs in different human IgG subclasses to evaluate the cross reactivity of human Fc against mCD16. The starting concentration for each mAb was 500 nM with a two-fold serial dilution.

**
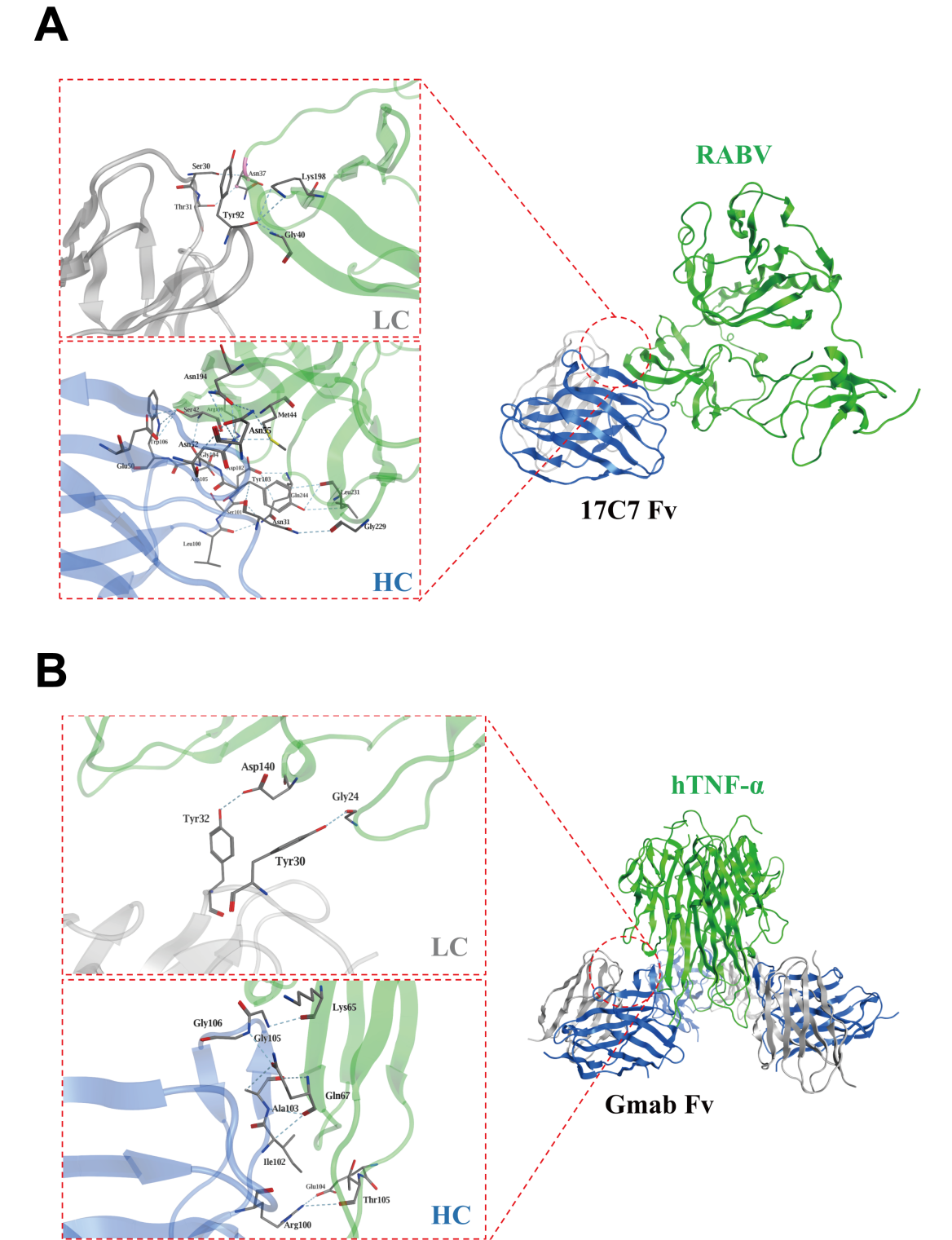
**

**Supplementary** Figure S4 Schematic diagram of the interaction residues between the candidate antibody variable domain and the target antigen.

1. Complex structure between the 17C7 variable domain and RABV (PDB: 8A1E).
2. Complex structure between the Gmab variable domain and hTNF-α. (PDB: 5YOY).

The red dashed box on the left highlights a magnified view of the interacting residues between the antibody variable domains and the antigen. Fv, variable region of the antibody heavy and light chains; HC, heavy chain variable domain; LC, light chain variable domain. Only interactions within a distance of 3.5 Å are labeled.


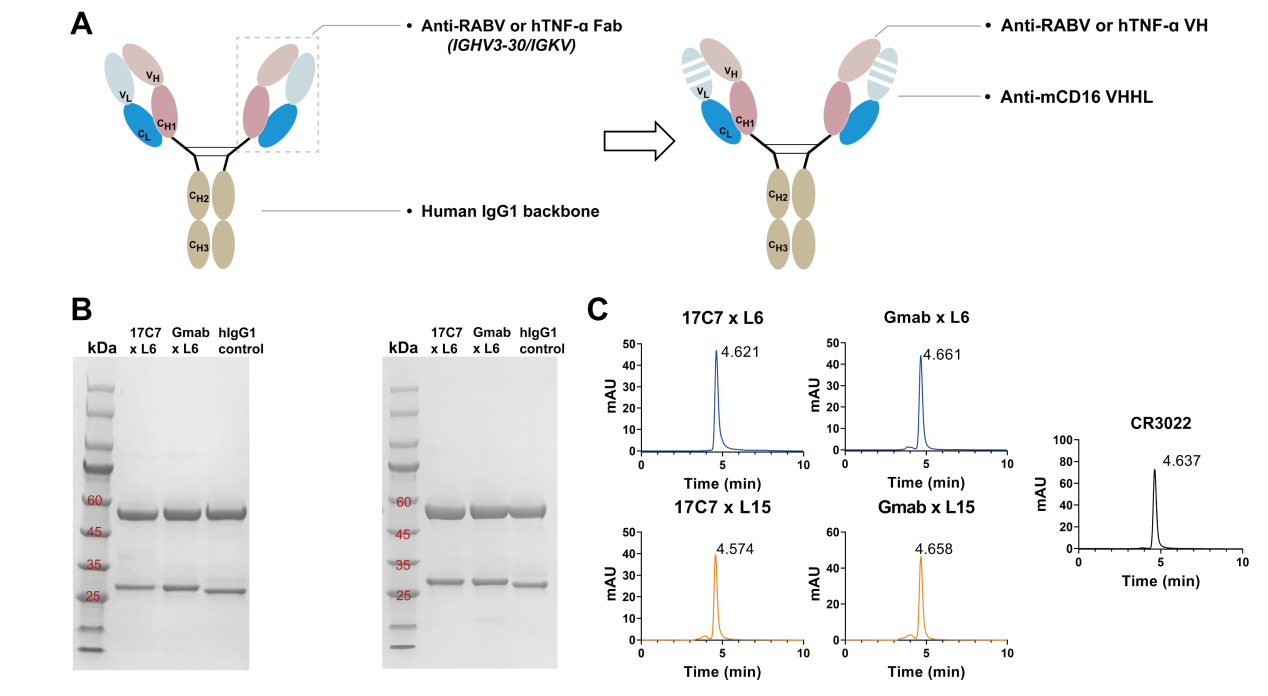


**Supplementary** Figure S5 Design and expression of IgG1-format bispecific antibodies based on modular replacement.

1. Schematic illustration of the novel IgG-format bsAb based on a modular replacement strategy.
2. SDS-PAGE demonstrates the purity of each hIgG1 bsAb.
3. Evaluation of the homogeneity of each hIgG1 bsAb by SEC-HPLC. In (B) and (C), A cross-SARS-reactive hIgG1 mAb, CR3022, was used as an isotype control.

**Supplementary** Table S1 The primer sets used for VHH amplification.

1. **VH + CH2 fragment amplification.**

| **Primer** | **Sequence** |
| --- | --- |
| Alpaca-F | GGTGGTCCTGGCTGC |
| Alpaca-R | GGTACGTGCTGTTGAACTGTTCC |

1. **VHH amplification.**

| **Primer** | **Sequence** |
| --- | --- |
| F1 | TACCGTGGCCCAGGCGGCCSARGTGCAGCTGGTGGAG |
| F2 | TACCGTGGCCCAGGCGGCCGAGGTGCRRCTGGTGGAG |
| F3 | TACCGTGGCCCAGGCGGCCSAGKTGCAGCTCGTGGAG |
| F4 | TACCGTGGCCCAGGCGGCCGAGGTGCAGKTSGTGGAG |
| F5 | TACCGTGGCCCAGGCGGCCCAGGTGCAGCTGGTAGAG |
| R1 | GGTGGTGGCCGGCCTGGCCYGAGGAGACRGTGACC |
| R2 | GGTGGTGGCCGGCCTGGCCTGAGGACACGGTGCCC |

**Supplementary Table S2 The primer sets used for amplification of diversity element CDRs and light chain backbone IGKV3-20.**

| **Target** | **Primer** | **Sequence** |
| --- | --- | --- |
| CDR1 | H1F-VL | TCCTGTGCAGCCTCTGG |
|  | H1R1-VL | AGCCTGGCCAGGTTTCTGCTGGACCCAGCYCAT |
|  | H1R2-VL | AGCCTGGCCAGGTTTCTGCTGGWACCAGCYCAT |
| CDR2 | H2F1-VL | AAACCTGGCCAGGCTCCCAGGCTCCTCATCTATGTCTCAGCTATT |
|  | H2F2-VL | AAACCTGGCCAGGCTCCCAGGCTCCTCATCTATGTGTCCRGTATT |
|  | H2R-VL | AGTGGCCCTGCTGTATGTGCTACC |
| CDR3 | H3F-VL | ATTTTGCCGTGTATTACTGT |
|  | H3R-VL | GCCGGCCTGGCCACTTTTGATTTCCACCTTGGTSCCCTGGCC |
| FR1 | ALLF-VL | GTTTCGCTACCGTGGCCCAGGCGGCCGAAATTGTGTTGACG |
|  | FR1R-VL | AGAGGCTGCACAGGAGAGGGTGGC |
| FR3 | FR3F-VL | GGTAGCACATACAGCAGGGCCACTGGCATC |
|  | FR3R-VL | ACAGTAATACACTGCAAAAT |

**Supplementary Table S3 The primer sets used for amplification of VHHL.**

| **Target** | **Primer** | **Sequence** |
| --- | --- | --- |
| FR1-CDR1  (Final-11) | ALLF-VL | GTTTCGCTACCGTGGCCCAGGCGGCCGAAATTGTGTTGACG |
|  | H1R1-VL | AGCCTGGCCAGGTTTCTGCTGGACCCAGCYCAT |
|  | H1R2-VL | AGCCTGGCCAGGTTTCTGCTGGWACCAGCYCAT |
| FR3-CDR3-FR4  (Final-33) | FR3F-VL | GGTAGCACATACAGCAGGGCCACTGGCATC |
|  | H3R-VL | GCCGGCCTGGCCACTTTTGATTTCCACCTTGGTSCCCTGGCC |
| FR2-CDR2-FR3-CDR3-FR4  (Final-23) | H2F1-VL | AAACCTGGCCAGGCTCCCAGGCTCCTCATCTATGTCTCAGCTATT |
|  | H2F2-VL | AAACCTGGCCAGGCTCCCAGGCTCCTCATCTATGTGTCCRGTATT |
|  | H3R-VL | GCCGGCCTGGCCACTTTTGATTTCCACCTTGGTSCCCTGGCC |
| VHHL | ALLF-VL | GTTTCGCTACCGTGGCCCAGGCGGCCGAAATTGTGTTGACG |
|  | ALLR-VL | GTCGCCGTGGTGGTGGTGGTGGTGGCCGGCCTGGCCACT |

**Supplementary Table S4 Amino acid selection in CDR1 under two different site-directed mutagenesis strategies.**

| **Position**  **(IMGT numbering)** | **Strategy 1** | **Strategy 2** |
| --- | --- | --- |
| H27 | G | G |
| H28 | F/G/R/S/Y | F/G/R/S |
| H29 | T | I/T |
| H30 | F | F/L |
| H35 | S | S |
| H36 | S/N/D/G | S/N/D/G |
| H37 | Y | Y |
| H38 | A/T/G/S | A/G |

**Supplementary Table S5 Amino acid selection in CDR2 under two different site-directed mutagenesis strategies.**

| **Position**  **(IMGT numbering)** | **Strategy 1** | **Strategy 2** |
| --- | --- | --- |
| H56 | I | I |
| H57 | S/N/T/Y | S/N/T |
| H58 | S/W | S/W |
| H59 | S/G/D/N | S/G/D/N |
| H62 | G | G/D |
| H63 | G | G |
| H64 | S/N/T/G/I/D/R | S/T/R/N/G/I |
| H65 | T | T |

**Supplementary Table S6 The primer sets for stepwise assembly of VHHL fragments via different site-directed mutagenesis strategies.**

| **Target** | **Primer** | **Sequence** |
| --- | --- | --- |
| FR1 | ALLF-VL | GTTTCGCTACCGTGGCCCAGGCGGCCGAAATTGTGTTGACG |
|  | FR1R | ACTGGCCCTGCAGGAGAGGGTGGC |
| CDR3 | OptiH3F-VL | GATTTTGCCGTNTAYTAYTGY |
|  | OptiH3R-VL | CCGGCCTGGCCACTTTTGATTTCCACCTTGGTNCCYTKGCCCCA |
| MCDR1  (Strategy 1 & Strategy 2) | F11 | TCCTGCAGGGCCAGTGGAVGTACCTTCAGTRRTTATRSTTTAGCCTGGTACCAG |
|  | F12 | TCCTGCAGGGCCAGTGGATWTACCTTCAGTRRTTATRSTTTAGCCTGGTACCAG |
|  | F21 | TCCTGCAGGGCCAGTGGAVGTAYAYTTAGTRRTTATGSATTAGCCTGGTACCAG |
|  | F22 | TCCTGCAGGGCCAGTGGATTTAYAYTTAGTRRTTATGSATTAGCCTGGTACCAG |
|  | FR2R-VL | ATAGATGAGGAGCCTGGGAGCCTGGCC |
| MCDR2  (Strategy 1 & Strategy 2) | F13 | AGGCTCCTCATCTATATCWMTTSGRRTGGCGGGRRTACCAGCAGGGCCACTGGC |
|  | F14 | AGGCTCCTCATCTATATCWMTTSGRRTGGCGGGABAACCAGCAGGGCCACTGGC |
|  | F23 | AGGCTCCTCATCTATATCAVTTSGRRTGRTGGGANTACCAGCAGGGCCACTGGC |
|  | F24 | AGGCTCCTCATCTATATCAVTTSGRRTGRTGGGRGAACCAGCAGGGCCACTGGC |
|  | FR3R-VL | RCARTARTANACGGCAAAAT |
| VHHL | ALLF-VL | GTTTCGCTACCGTGGCCCAGGCGGCCGAAATTGTGTTGACG |
|  | ALLR-VL | GTCGCCGTGGTGGTGGTGGTGGTGGCCGGCCTGGCCACT |

**Supplementary Table S7 Interaction residues between the mAb 17C7 heavy chain variable region and RABV antigen.**

| **Interaction residues (Ab-Ag)** | **Interaction distance（Å）** | **Interaction types** |
| --- | --- | --- |
| Asn31-Gly229 | 2.93 | Hydrogen bond |
| Glu50-Ser42 | 3.19 | Hydrogen bond |
| Asn52-Met44 | 2.88 | Hydrogen bond |
| Asn52-Asn194 | 2.74 | Hydrogen bond |
| Asn55-Met44 | 3.89 | Hydrogen bond |
| Asn55-Asn194 | 3.03 | Hydrogen bond |
| Leu100-Gln244 | 3.27 | Hydrogen bond |
| Ser101-Gln244 | 3.43 | Hydrogen bond |
| Asp102-Arg199 | 3.35 | Ionic bond |
| Asp102-Met243 | 3.96 | Hydrogen bond |
| Asp102-Gln244 | 2.93 | Hydrogen bond |
| Tyr103-Leu231 | 2.98 | Hydrogen bond |
| Gly104-Ser42 | 3.11 | Hydrogen bond |
| Asp105-Phe41 | 3.64 | Hydrogen bond |
| Asp105-Arg199 | 3.12 | Ionic bond |
| Trp106-Ser42 | 3.13 | Hydrogen bond |

Use the protein-protein interaction analysis function built into MOE software to analyze the interaction sites between antibodies and target antigens in candidate structures.

**Supplementary Table S8 Interaction residues between the mAb Gmab heavy chain variable region and hTNF-α antigen.**

| **Interaction residues (Ab-Ag)** | **Interaction distance（Å）** | **Interaction types** |
| --- | --- | --- |
| Ser31-Glu110 | 3.82 | Hydrogen bond |
| Tyr53-Glu110 | 3.67 | Aromatic interaction |
| Arg100-Glu104 | 3.04 | Ionic bond/  Hydrogen bond |
| Arg100-Thr105 | 3.44 | Hydrogen bond |
| Arg100-Glu107 | 3.64 | Ionic bond/  Hydrogen bond |
| Arg100-Ala111 | 3.68 | Hydrogen bond |
| Gly101-Gln67 | 3.56 | Hydrogen bond |
| Ile102-Gln67 | 3.13 | Hydrogen bond |
| Ala103-Gln67 | 3.12 | Hydrogen bond |
| Gly105-Lys65 | 2.68 | Hydrogen bond |
| Gly106-Gln67 | 3.07 | Hydrogen bond |
| Tyr108-Gly24 | 3.71 | Hydrogen bond |

Use the protein-protein interaction analysis function built into MOE software to analyze the interaction sites between antibodies and target antigens in candidate structures.
